# Within- and between-brood variations in haemosporidian infections of nestlings reveal complex host–vector–parasite relationships

**DOI:** 10.1007/s10336-026-02417-1

**Published:** 2026-06-03

**Authors:** Anna George, Christian Aastrup, Tamara Emmenegger, Arne Hegemann

**Affiliations:** 1 Department of Biology, Lund University, Ecology Building (Sölvegatan 37), 223 62 Lund, Sweden; 2 Department of Zoology, Museum Lucerne, Kasernenplatz 6, 6003 Lucerne, Switzerland; 3 Institute of International Animal Health/One Health, Friedrich-Loeffler-Institute, Federal Research Institute for Animal Health, Südufer 10, 17493 Greifswald, Insel Riems Germany

**Keywords:** Haemosporidia, Avian malaria, Host–parasite interaction, Life history, Ontogeny

## Abstract

Avian blood parasites are vector-transmitted protozoan parasites and common in wild birds. They cause acute and chronic infections at different life stages with potentially detrimental effects on host fitness. Transmission often happens during the nestling period, when biting dipterans have easy access to featherless skin. However, little is known about patterns of infection within and between broods. Therefore, we investigated prevalence of haemosporidians (*Leucocytozoon, Plasmodium* and *Haemoproteus*) in Jackdaw (*Corvus monedula*) nestlings over four non-consecutive years. Prevalence varied by year, averaged at 33.9%, with *Leucocytozoon* being the most prevalent genus. We found no links between infection and morphometry, nor with sex or hatching date, at the population level. However, at the brood level, among female nestlings, those with shorter tarsi were more likely to be infected than female siblings with longer tarsi. Infection probability was positively correlated with brood size at the brood level, but not at the individual level. Finally, more broods were partially infected than fully infected or uninfected. Thus, the observed patterns in prevalence were manifold, indicating that haemosporidian infections, caused by bites of vectors, are influenced by various factors in this host–vector-parasite system.

**Supplementary Information:**

The online version contains supplementary material available at 10.1007/s10336-026-02417-1.

## Abstract (de)

**Variation bei Hämosporidieninfektionen von Nestlingen innerhalb und zwischen Bruten zeigt komplexe Wirts-Überträger-Parasiten-Beziehungen**

Aviare Blutparasiten (Hämosporidien) werden durch blutsaugende Insekten (z.B. Stechmücken, Gnitzen, Lausfliegen, Kriebelmücken) übertragen und sind in wildlebenden Vögeln weit verbreitet. Sie verursachen akute und chronische Infektionen in verschiedenen Lebensstadien und können potenziell nachteilige Auswirkungen auf die Fitness des Wirts haben. Die Übertragung erfolgt häufig während der Nestlingsphase, wenn stechende Dipteren leichten Zugang zu unbefiederter Haut haben. Über Infektionsmuster innerhalb und zwischen Bruten ist jedoch bislang wenig bekannt. Vor diesem Hintergrund analysierten wir das Vorkommen von Hämosporidien (*Leucocytozoon, Plasmodium* und *Haemoproteus*) bei Dohlen (*Corvus monedula*) im Nestlingsstadium über vier nicht aufeinanderfolgende Jahre. Die Häufigkeit von Infektionen variierte zwischen den Jahren und lag im Mittel bei 33,9%, wobei *Leucocytozoon* die häufigste Gattung darstellte. Auf Populationsebene ergaben sich keine signifikanten Zusammenhänge zwischen Infektionsstatus und morphometrischen Merkmalen sowie kein Effekt von Geschlecht oder Schlupfdatum. Auf Brutebene zeigte sich jedoch, dass unter weiblichen Nestlingen Individuen mit kürzeren Tarsen mit höherer Wahrscheinlichkeit infiziert waren als ihre weiblichen Geschwister mit längeren Tarsen. Zudem war die Infektionswahrscheinlichkeit auf Brutebene positiv mit der Brutgröße assoziiert, während ein entsprechender Zusammenhang auf Individualebene nicht nachweisbar war. Außerdem waren mehr Bruten partiell infiziert als vollständig infiziert oder vollständig frei von Infektionen. Die beobachteten Prävalenzmuster waren somit vielfältig und weisen darauf hin, dass Hämosporidieninfektionen, die durch Vektorstiche übertragen werden, in diesem Wirt-Überträger-Parasit-System von verschiedenen Faktoren beeinflusst werden.

## Introduction

Parasites are ubiquitous in nature and patterns of parasite prevalence in populations of free-living animals can provide interesting insights into the evolutionary ecology of hosts and their parasites (Poulin 2007). The individual risk of becoming infected by parasites is determined by a combination of factors, including local parasite abundance, the probability of hosts to encounter parasites, and the hosts’ immunocompetence (Schmid-Hempel 2021). When a vector is involved in parasite transmission, an additional link is added to the chain of events leading to the individual infection status of a host.

Avian haemosporidians are well-studied, vector-transmitted blood parasites (Marzal 2012). These protozoan parasites are transmitted via the bite of dipteran insects (Valkiūnas 2005), specifically biting midges (Ceratopogonidae), louse flies (Hippoboscidae), black flies (Simuliidae), and mosquitoes (Culicidae). While the prevalence patterns of adult birds have been widely studied in passerine species (e.g. Ellis et al. 2020; Popescu et al. 2020), the studies examining these infections in nestlings have been scarce and often found low prevalences (e.g. Dunn et al. 2017; Cosgrove et al. 2006). Yet, transmission is hypothesised to often happen during the nestling period (López-Rull and Macías Garcia 2015), because vectors can easily access the featherless skin of young nestlings and because nestlings are less mobile making it difficult to defend themselves against biting insects. Nevertheless, little is known about the relative importance of factors influencing the nestlings’ attractivity to vectors of haemosporidian parasites (Gutiérrez-López et al. 2019; Yan et al. 2017).

Despite high infection risk during the nestling period, many studies on nestlings report seemingly low prevalences. This can potentially be explained by the time it takes parasites to reach the host’s peripheral bloodstream after transmission. The typical length of this prepatent period ranges from 7 days to 2 weeks and varies between host species, haemosporidian genera and lineages (Valkiūnas 2005). Hence, nestlings often fledge before the prepatent period is complete, and studies on nestlings’ blood parasites are mainly restricted to species with long nestling periods, such as raptors, owls, or pigeons (Ashford et al. 1991; Svobodová et al. 2015; Dunn et al. 2017; Chakarov et al. 2008, 2020). Thus, our knowledge of infection prevalence in passerine nestlings is still limited.

When comparing broods, it has been hypothesised that large broods have higher risks of being infected, as they produce more CO_2_ (a potential attractant for dipteran vectors; see Marzal et al. 2022). However, prevalence in Sparrowhawk (*Accipiter nisus*) and Common Buzzard (*Buteo buteo*) nestlings was found to be only weakly or not at all associated with brood size both at the individual and brood level (Svobodová et al. 2015; Chakarov et al. 2008). Another study on several passerine species, however, found higher abundances of haemosporidian vectors in nests with large broods (Martínez-de la Puente et al. 2009). Furthermore, vector abundances vary over the year due to seasonal changes in ambient conditions (Martínez-de la Puente et al. 2009) causing infection prevalence to vary between broods according to hatching date. Accordingly, later hatched broods of sparrowhawks and buzzards were found to have higher prevalence of *Leucocytozoon* and *Haemoproteus* infections compared to early hatched broods (Svobodová et al. 2015). To shed light on this, infection prevalences need to be explored across the full breeding season, ideally for several haemosporidian genera and at the lineage level.

At the within-brood level, the tasty chick hypothesis for ectoparasites, developed by Christe et al. (1998), suggests that the within-brood rank of nestling size could be an important factor influencing parasitism. Christe et al. (1998) proposed that larger nestlings tend to have a more well-developed immune function than smaller siblings and hence better defence against pathogens. Consequently, parasites may infect smaller nestlings, benefiting other siblings and the parents by improving their overall reproductive success. While some studies support the tasty chick hypothesis for ectoparasites (e.g. Simon et al. 2003, for blow flies, and Wemer et al. 2021, for carnid flies), overall evidence is mixed across bird and parasite taxa (Roulin et al. 2003; Brown and Brown 2018; Valera et al. 2004). Alternative hypotheses suggest that bigger individuals in good condition are preferred targets for vectors, because they have a larger surface area for biting, provide better nutritional value, and allow easier access to blood due to good circulation (Valera et al. 2004). To disentangle the relative importance of the tasty chick hypothesis compared to alternative hypotheses for avian blood parasites is challenging and has, to the best of our knowledge, not yet been evaluated.

In this study, we explore blood parasite prevalence over four non-consecutive years (2014, 2015, 2018, and 2022) in nestlings of Eurasian Jackdaws (*Corvus monedula;* hereafter referred to as Jackdaws), as their particularly long nestling period (32–35 days; Aastrup and Hegemann 2021) allows infections to reach the peripheral bloodstream before fledging. First, we explore the haemosporidian parasite prevalence across years, describe which haemosporidians are found and whether mixed infections occur (Q1). We then investigate the lineage diversity and also examine the within-brood lineage composition (Q2). We test whether brood size can explain infection status both at the brood and the individual level (Q3). At the brood level, we investigate how often broods are partially infected and how often all members of broods are infected or uninfected and if those patterns differ among years (Q4). At the individual level, we test whether infection status differs between year, sampling date or sex (Q5). To further shed light on individual infection patterns, we test three morphometric measurements (mass, wing length and tarsus length) as potential explanatory factors for variation of infections at the population level (Q6). In addition, we calculate growth rates to test if infected individuals grow more slowly than uninfected individuals (Q7). To evaluate the tasty chick hypothesis, we use only partially infected broods and explore whether siblings of different size ranks differ in infection risk (Q8). Finally, we compare the infections of parents and their nestlings for one of the study years (2015), to check for potential indications about the pathways of transmission (Q9), in particular if within-family transmission of blood parasites from parent to offspring might occur (cf. Chakarov et al. 2015).

## Materials and methods

### Study area and species

The study took place in a nest box colony of jackdaws in Revingehed (55° 43′ 1.938″ N, 13° 26′ 22.477″ E), in Southern Sweden. This small, wooded study site, consisting of deciduous trees and pine, is surrounded by extensively grazed pastures on a military field (see also Aastrup and Hegemann 2021; Aastrup et al. 2023). The maximum distance between nest boxes is ca. 190 m in east–west direction and ca. 80 m in north–south direction. The 63 nest boxes used in this study are roughly equally distributed over the area; however, some clustering with distances of ca. 3 m between nest boxes occurs depending on the availability of suitable trees (for a map of the colony with nest box distribution, see Supplementary material). Occupancy rate is usually close to 100% (see Aastrup et al. 2023). Nest boxes are fixed in trees at approximately 3–4 m above the ground (for further details on nest box design, see Aastrup et al. 2023 and Supplementary material). Jackdaws are a cavity-nesting corvid that can breed in colonies and raise a single brood each year. In all years, we started to check nest boxes from mid-April every third day to determine laying of the first egg and clutch size. To determine day of hatching, we checked nest boxes daily from day 17 after the first egg was laid. This allowed us to determine exact age, i.e., days 12 and 29 (see below). Jackdaws have asynchronous hatching, but in a previous study (Aastrup et al. 2023), we show that at 12 days of age only 3.3% of broods have an age variation of up to 2 days. We therefore calculated the date at which a brood would be 12 or 29 days of age from the first hatching and pooled the age of all nestlings in the brood (see also Aastrup and Hegemann 2021; Aastrup et al. 2023). In our colony, the nestling period is 32–35 days and the average brood size at day 29 is 2.7 (Aastrup and Hegemann 2021, Hegemann unpublished data). In jackdaws, only females incubate and brood, but both parents provide food for the nestlings from hatching onwards.

### Blood sampling and body measurements

We investigated blood samples from a total of 374 nestlings, from 150 broods, across four breeding seasons, 2014 (*n* = 93 nestlings/35 broods), 2015 (*n* = 84/37), 2018 (*n* = 142/57), and 2022 (*n* = 55/21). We took biometric measurements when nestlings were 29 days old, while in 2018, the same biometric measurements were also taken at 12 days of age (Aastrup et al. 2023), allowing the calculation of growth rates for nestlings in 2018. We measured tarsus length to the nearest 0.1 mm using callipers, wing length to the nearest mm using a wing ruler and body mass to the nearest 0.1 g using a digital scale. Each year, nestlings were sampled at 29 days old (between 2nd and 24th June) to maximise the chance that parasites can reach the peripheral blood after the prepatent period, while avoiding premature fledging due to handling. In 2014, we also blood sampled some adult birds caught in the nest box when feeding their nestlings. The number of nestlings in each brood was recorded as the number present on the day of blood sampling, excluding any nestlings that may have been present earlier but died any time before this day.

Blood was sampled by venipuncture of the brachial vein using sterile hypodermic needles and heparinised capillaries and was then stored in 500 μl SET buffer (0.15 M NaCl, 0.05 M Tris, 0.001 M EDTA, adjusted to pH8 with NaOH). Samples were stored on ice until later the same day, when samples were placed in a freezer at – 49 °C until DNA extraction. Blood smears were not made.

### DNA extraction

DNA extraction was carried out using a standard ammonium acetate protocol (for details, see Ventura et al. 2025). In brief, blood samples in SET buffer were defrosted and vortexed, and then, 125 μl was transferred into a new tube and 3.5 μl of 20% SDS and 2.5 μl of 20 mg/ml proteinase K were added. After shaking samples, they were incubated for about 17 h in a water bath at 56 °C. The following day, we added 125 μl of 4 M ammonium acetate (NH_4_Ac). Samples were then cooled to room temperature, shaken well, and left incubated at room temperature for 1 h, during which they were shaken every 15 min. We then centrifuged at 13000 rpm for 15 min before we transferred the supernatant into a new tube. After adding 500 μl ice cold ethanol, we shook the samples again before centrifuging at 13000 rpm for 15 min. Afterwards, the supernatant was poured off and discarded, and only the DNA pellet was contained. Then, we added 250 μl ice cold 70% ethanol and carefully discarded immediately. Afterwards, the samples were left without lids at room temperature at least overnight (longer if necessary) to air dry completely. Finally, we added 20 μl of 1 × TE buffer and vortexed samples before storing them in the fridge for 3 days. The concentration of extracted DNA was quantified using a Nanodrop and then diluted with 1 × TE buffer to a concentration of 25 ng/μl. Molecular sexing for nestlings from 2014, 2015, and 2018 had been done previously for another study (see Aastrup and Hegemann 2021) according to the method in Richardson et al. (2001). Nestlings from 2022 were not molecularly sexed.

### Multiplex PCR

We used a multiplex polymerase chain reaction (PCR) to simultaneously screen for *Leucocytozoon* (L), *Plasmodium* (P), and *Haemoproteus* (H) infections (Ciloglu et al. 2019). We used one negative control (double distilled water) for every 22 samples, and one positive control of each of the three parasite genera (L, P, H) for every 43 samples. Gel electrophoresis was carried out with 2.5 μl of the PCR products on 2% agarose gel containing GelRed gel stain following Ciloglu et al. (2019), with the modifications of running at 80 V for 25 min instead of at 90 V for 1 h. We distinguished parasite genera based on fragment length and gels were imaged using a BioRad camera.

### Nested PCR 1 and 2

Samples positive for one or more parasite genera in the multiplex PCR were run in a two-step nested PCR to obtain the standard sequences for lineage determination following Hellgren et al. (2004) with minor modifications according to our lab standards: For all samples, we changed the cyclic reaction at 94 °C to 2 min (instead of 3 min) in PCR 1 and PCR 2 (for 22 cycles instead of 20 in PCR 1) and we checked nested PCR by running 2.5 μl (instead of 1.5 μl) of the PCR products from nested reaction 2 with 1 μl of stop-mix on a 2% agarose baby gel at 80 V for 25 min.

### Precipitation of PCR products and sequencing

The products from the nested PCR 2 were then precipitated with ammonium acetate (NH₄Ac) in preparation for the sequencing reaction. For the sequencing reaction, we used the forward and reverse primers to resolve full sequences. The PCR products from the sequencing reaction were then precipitated with EDTA. The samples were then sequenced on an ABI 3100 machine. After this, we aligned the sequences bi-directionally against reference sequences for parasite cytochrome b lineages known to be found in jackdaws, using the software BioEdit Sequence Alignment Editor (Hall 1999). All consensus sequences were finally compared to the known parasite lineages in the MalAvi database (Bensch et al. 2009, version 2.5.8, accessed 27th June 2023). If apparent double infections occurred in the chromatograms, we used a conservative approach: If the dominant peak of double peaks in the chromatogram corresponded to a known lineage from the MalAvi database, we attributed this lineage to be involved in this infection.

### Data analysis

Of the 374 screened nestlings, 5 nestlings from 5 different broods were excluded from further analysis as they gave ambiguous results when the PCR products were run on the gel (i.e. band position or band width did not match the control). For the other 369 individuals (from 150 broods), we obtained unambiguous positive or negative infection states. Data analysis was carried out in R version 4.1.2 (R Core Team 2024).

To answer Q1, we calculated the percentage of infected individuals per genus and furthermore compared prevalence between years using Pearson’s Chi-squared test. To answer Q2, we described the detected linages (descriptive, no statistics applied). To test if brood size relates to infections (Q3), we used a generalised linear model (GLMM) with logit link and binomial distribution using function *glmer* from package *lme4* (Bates et al. 2015). Infection status (infected/not infected) per brood and individual respectively was used as dependent variable and brood size as explanatory variable. Nest box nested within year was included as a random factor to account for non-independence of nestlings from the same brood. To answer Q4, we followed the method of Svobodová et al. (2015) on Common Buzzards and Sparrowhawks, and categorised broods as infected if at least one nestling was infected, and as uninfected if no nestling was infected. We compared the occurrence among years using a Pearson’s Chi-squared test and to test whether the occurrence of fully, partially, or none-infected broods differed from random, we applied Chi-squared goodness-of-fit tests. For these analyses, we had to exclude broods with only one nestling (*n* = 22 broods), since those broods cannot be partially infected. Likewise, we had to exclude broods where the infection status of one or more nestlings was unknown (*n* = 9 broods), since it requires infection status for all nestlings to know in which category a brood falls. Thus, 119 broods remained in the dataset for this analysis.

For the analysis on the individual-level and to explore whether year, sampling date, or sex relate to infection (Q5), we also used a GLMM as for Q3. We included the interaction between sampling date and year. The explanatory variable year was scaled and the variable sampling date was centred. For this analysis, we removed individuals with unknown infection status but kept their siblings. Year was set as a factor with 4 categorical levels. One brood of 2 nestlings in 2018 was removed from the dataset as it took place much later than all other broods in any year (8 day gap to the second last brood; Jackdaws within the colony are highly synchronised and otherwise the largest gaps is a max of 1 day; thus, this late brood was very likely a replacement clutch).

To explore whether body measurements at day 29 relate to infection status (Q6), we used similar GLMM as for Q3 and Q5, and used body mass, wing length, and tarsus length as explanatory variables. To explore whether infected nestlings might grow at different speeds compared to uninfected individuals (Q7), we used the same model structure as for Q6 but with the growth rates (expressed as delta measurements between day 12 and day 29 for mass, wing length, and tarsus length; only available for 2018, see above) as explanatory variables instead. Even though we do not know the infection status at day 12, the relatively long prepatent period of the parasite presumes that nestlings infected at day 29 must have acquired their infection earlier during the nestling phase, likely before or shortly after day 12. Hence, comparing growth rates between day 12 and 29 of age of nestlings that we detected positive at day 29, likely composes a comparison of growth rates between infected and uninfected birds, though some uncertainty about the exact time of infection and the stage of infection remains (see also Rinaud et al. 2025).

To evaluate the tasty chick hypothesis, which relates to within-brood variation in nestling size, we analysed individual size rank in relation to infection status (Q8). For this, nestlings were ranked following a method adapted from Ashford et al. (1991) and Brown and Brown (2018), resulting in rank 1 for the largest, rank 3 for the smallest, and rank 2 for all intermediate members of the brood. If two nestlings were very close in a biometric measurement, they could receive the same rank. Based on the spread of data within each metric, we classified nestlings as same rank for wing length, if they differed by less than 2 mm (1% based on average length), for body mass when they differed by < 1 g (0.5% based on average body mass), and for tarsus length if they differed by < 0.2 mm (0.4% based on average length). We only included partially infected broods (i.e. broods with at least 1 but less than all nestlings were infected, *n* = 161 nestlings, 54 broods) and excluded broods in which all nestlings were similar in size, so that they could not be separated in different ranks, leaving a sample size of *n* = 141 nestlings, from 47 broods. We used the similar model structure as for Q3, 5, 7 and included the rank for each biometric measurement as well as the two-way interactions with sex, but used the *glm* function from base R and hence no random effect. This was necessary, because due to too few observations for many of the levels, we could not include nest box nested in year as random factor as the model would not converge. For models containing the body size measurements (Q6), we confirmed that there was no problem of multicollinearity among factors, since the variance inflation factor (VIF function in the *car* package; Fox and Weisberg 2019) was < 2 for all three variables.

For the comparison of infections of adults and their nestlings (Q9), we descriptively collated the infection status, parasite genus and lineage of adults and their corresponding nestlings.

For all models, we always started with a full model and used the *drop1* function with Chi-squared test, from base R, to determine in which order to remove non-significant factors from the model, using a significance level of *p* < 0.05.

## Results

### Parasite prevalence (Q1)

**Fig. 1:**
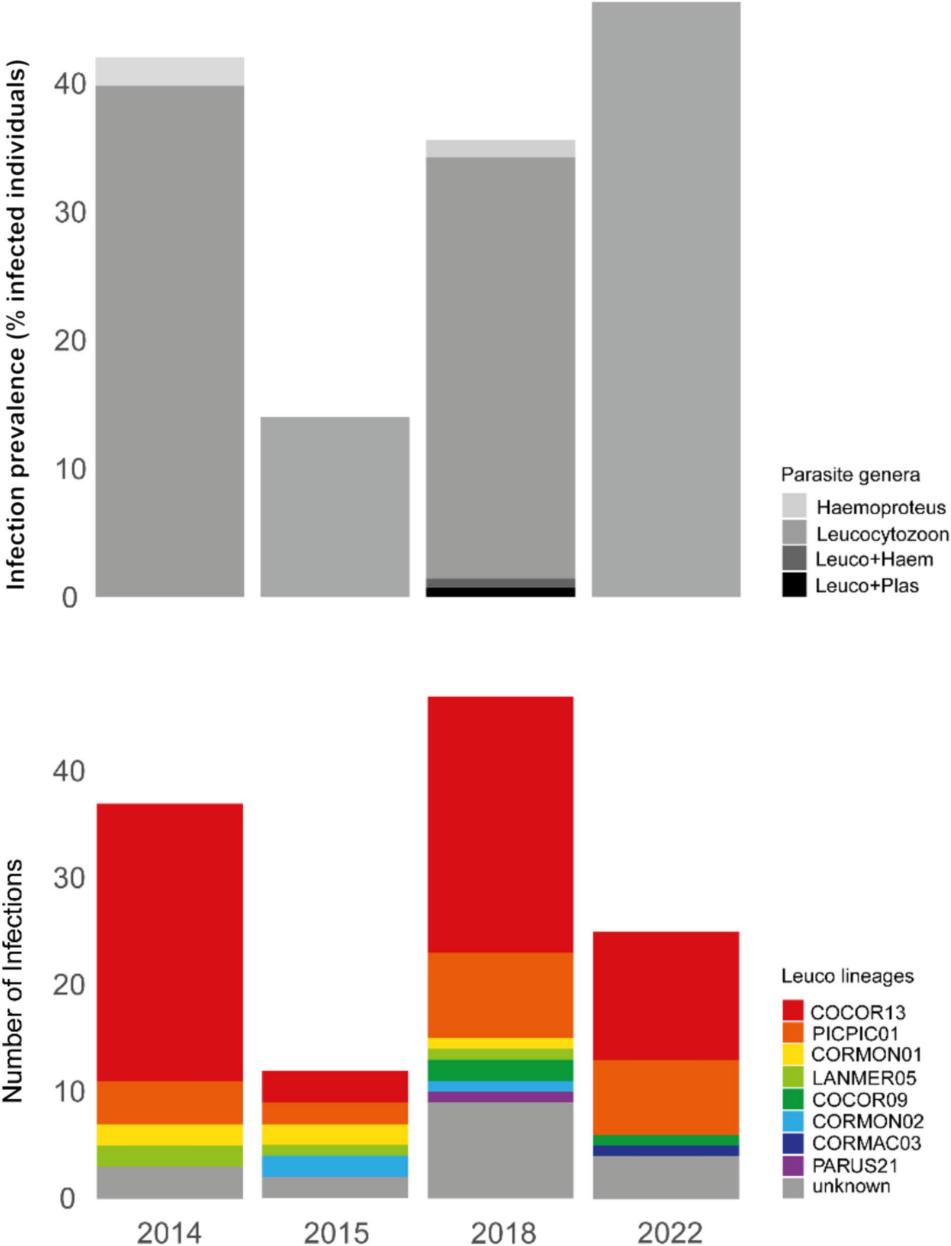
Annual haemosporidian prevalence of 369 jackdaw nestlings from 150 broods from a population in southern Sweden by parasite genus (upper panel) and separate for lineages of the genus *Leucocytozoon* (lower panel). 2014: n = 93 nestlings / 35 broods, 2015: n=83/37, 2018: n=139/56, 2022 n=54/21

A total of 125 of 369 sampled individual nestlings (33.9%) were infected with at least one genus of haemosporidian parasite across the 4 years in the study. Of these 125 infections, 119 were single *Leucocytozoon* infections (95.2%), 4 were single *Haemoproteus* infections (3.2%), and no single *Plasmodium* infection was found. We found two mixed infections, of which one comprised *Leucocytozoon* and *Plasmodium* (0.8%), and one *Leucocytozoon* and *Haemoproteus* (0.8%). At the brood level, 78 out of 146 broods (53.4%) had at least one infected nestling. Parasite prevalence varied significantly between years (Pearson’s Chi-squared test, *χ*^2^ = 20.49, df = 3, *p* < 0.001). Prevalence was highest in the year 2022 at 46.3%, and lowest in 2015 at 14.5%. With 35.3% and 41.9%, respectively, prevalence was intermediate in 2018 and in 2014 (Fig. 1, top panel).

### Lineage diversity and composition (Q2)

We assigned 110 parasite sequences, from 107 individuals to 11 different haemosporidian lineages: 8 *Leucocytozoon* (Fig. 1), 1 *Plasmodium* (SGS1), and 2 *Haemoproteus* (ROFI1 and WW2). Another eighteen samples did not return full-length sequences. The partial sequences suggest that these belong to L*eucocytozoon*, but the lineages had to be classified as ‘unknown’. The *Leucocytozoon* lineages COCOR13 and PICPIC01 were present in all four study years, with COCOR13 being the most prevalent lineage in every study year, and PICPIC01 being the second most prevalent in all years, except in 2015, when PICPIC01, CORMON01 and CORMON02 were equally frequent (Fig. 1, bottom panel).

We also examined the within-brood haemosporidian lineage composition. For fully infected broods with known infection status and lineage for all nestlings in the brood (*N* = 9 broods), nestlings were infected with the same lineage as their sibling in four broods, of which all were broods of two nestlings each. In the other five broods, which were broods of three nestlings (*n* = 3), four nestlings (*n* = 1), or two nestlings (*n* = 1), nestlings were infected with different lineages from their siblings. However, always two nestlings in each brood were infected with the same lineage, except in the brood of two.

### Brood size and infection status at the brood and individual level (Q3)

**Fig. 2:**
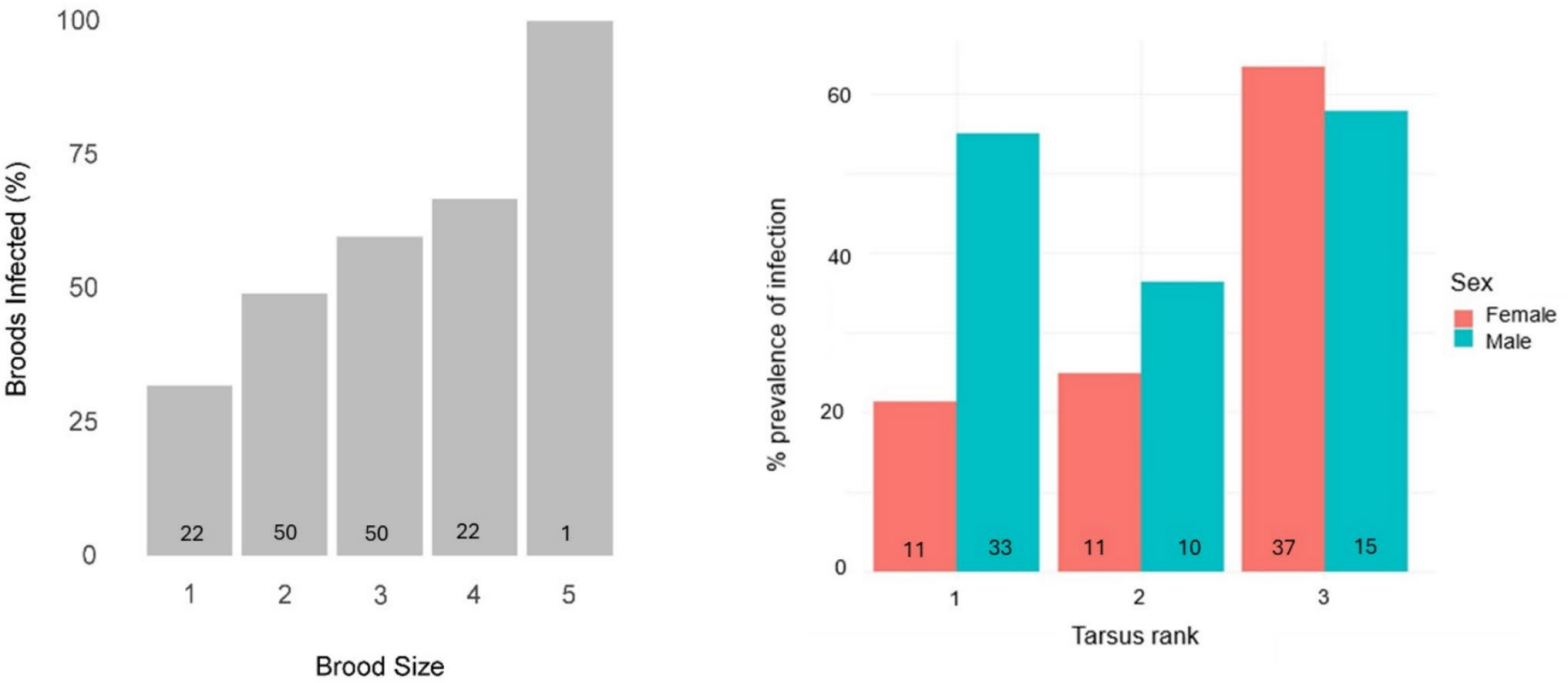
Proportion of jackdaw broods from a Swedish study population infected with haemosporidians in relation to brood size (left panel) and the haemosporidian prevalence in relation to sex and the tarsus length rank (1 = longest tarsus, 2 = intermediate tarsi, 3 = shortest tarsus) within the brood (right panel). Data from four non-consecutive study years. Numbers in bars reflect sample sizes

Brood infection status was positively related to brood size (Fig. 2, left panel). This held true both when including the only brood with five nestlings (GLMM, Wald *χ*^2^ = 4.28, df = 1, *p* = 0.039, *n* = 145) and when excluding it (GLMM, Wald *χ*^2^ = 3.94, df = 1, *p* = 0.047, *n* = 144). In contrast, the infection status of individual nestlings was not significantly related to brood size (GLMM, Wald *χ*^2^ = 0.013, df = 1, *p* = 0.91, *n* = 367).

### Fully, partially, and none-infected broods (Q4)

**Fig. 3:**
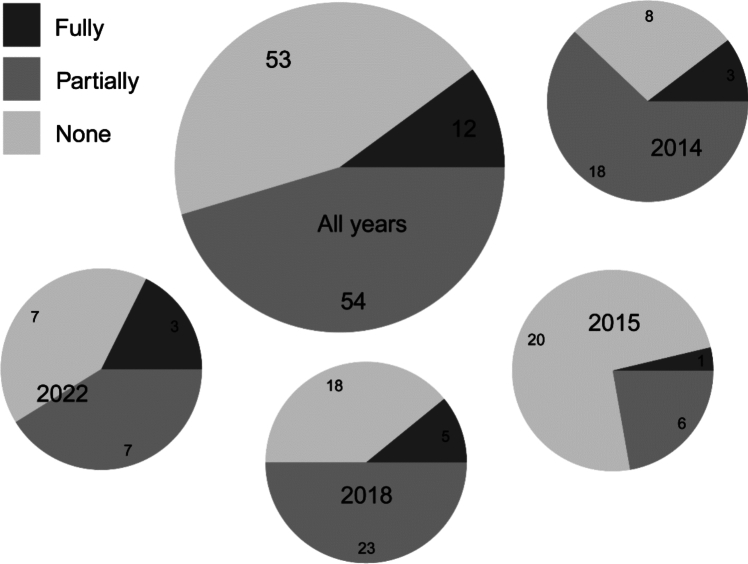
Proportions of jackdaw broods from a Swedish study population fully, partially, or completely uninfected with haemosporidian parasites for all years (big circle) and for each of the study years separately (small circles). Sample sizes, as number of broods, are given in each pie chart

The proportions of broods that were fully, partially, and none-infected differed significantly between years (Fig. 3; Pearson’s Chi-squared test, *χ*^2^ = 15.489, df = 6, *p* = 0.017). This pattern was driven by the low prevalence in 2015, as, when 2015 was removed from the analysis, there was no significant difference in proportions between years (Pearson’s Chi-squared test, *χ*^2^ = 2.728, df = 4, *p* = 0.6). The observed proportions of fully, partially, or none-infected broods differed from the expected proportions (if infections were distributed randomly) when all years were grouped together (Chi-squared goodness-of-fit test *χ*^2^, *p* < 0.001) and in all years except 2022 (*χ*^2^, 2014, *p* = 0.002; 2015, *p* < 0.001; 2018, *p* = 0.004; 2022, *p* = 0.390). The proportion of broods that were partially infected was higher than (in 2014 and 2018) or equal to (in 2022) the proportion of broods in which none were infected, in all years except in 2015, when the overall prevalence was low and the proportion with none infected was the highest (Fig. 3).

### Year, sampling date, and sex (Q5)

Nestling infection status was not significantly related to sampling date, year, and their interaction when included in a GLMM together (Date, Wald *χ*^2^ = 0.37, df = 1, *p* = 0.54, *n* = 367; year, Wald *χ*^2^ = 1.33, df = 1, *p* = 0.25, *n* = 367; year: date, Wald *χ*^2^ = 0.82, df = 1, *p* = 0.36, *n* = 367). Infection status was marginally related to year when used alone in a GLM (LR *χ*^2^ = 3.2, df = 1, *p* = 0.07). With an infection rate of 32.5% in males and 31.8% in females, nestling sex was not significantly related to infection status (GLMM, Wald *χ*^2^ = 1.52, df = 2, *p* = 0.47, *n* = 311).

### Body measurements at day 29 (Q6) and growth rates from day 12 to day 29 (Q7)

**Fig. 4:**
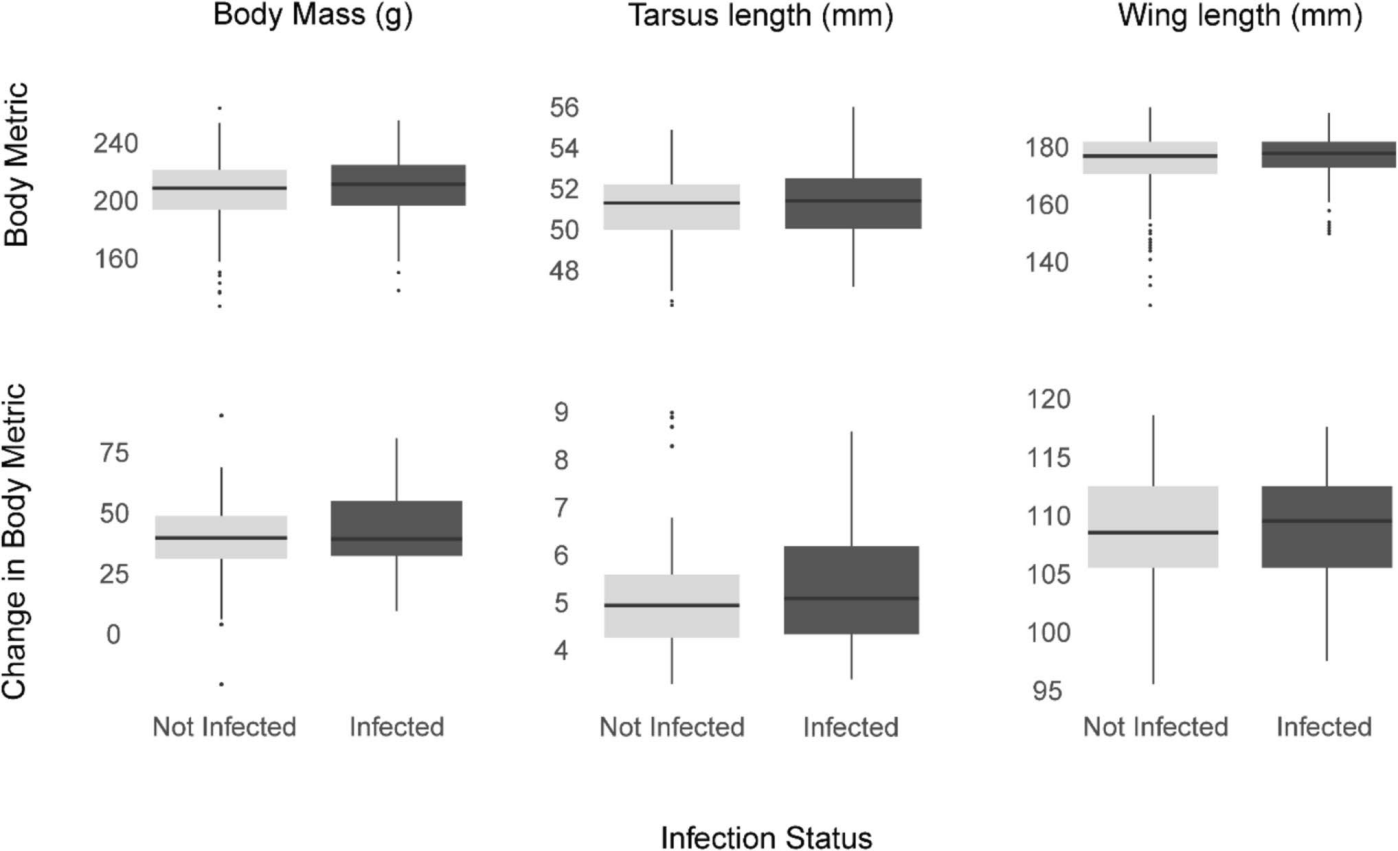
The absolute body metrics at day 29 (top row, n= 369 jackdaw nestlings from 150 broods in any of the four study years 2014, 2015, 2018 and 2022) and the change in those body metrics between day 12 and 29 (bottom row, n=142 jackdaw nestlings from 57 broods, only 2018, see methods) infected or not infected with haemosporidian parasites (points represent outlying values). Birds were sampled in a Swedish study population. Please observe that the body metric (i.e. body mass, tarsus length, and wing length) is labelled on the top of each column

Body mass, wing length, and tarsus length were all not significant predictors of nestling infection status (Fig. 4, top row: GLMM, body mass, Wald *χ*^2^ = 1.31, df = 1, *p* = 0.25, *n* = 367; wing length, Wald *χ*^2^ = 0.25, df = 1, *p* = 0.618, *n* = 367; tarsus length, Wald *χ*^2^ = 0.01, df = 1, *p* = 0.92, *n* = 367).

Growth rates of nestling body mass, wing length, and tarsus length were not significantly correlated with infection status (Fig. 4, bottom row: GLMM, *Δ* body mass, Wald *χ*^2^ = 0.36, df = 1, *p* = 0.55; *Δ* wing, Wald *χ*^2^ = 0.34, df = 1, *p* = 0.56; Δ tarsus, Wald *χ*^2^ = 0.72, df = 1, *p* = 0.39, *n* = 125 nestlings, 50 broods).

### Size rank and infection: the tasty chick hypothesis (Q8)

Using only partially infected broods (*n* = 47) and nestlings that differed from their siblings enough to rank them in that metric (body mass, wing length or tarsus—see the section “Methods”), the rank according to wing length did not have a significant relation to nestling infection status (GLM, wing length rank, LR *χ*^*2*^ < 0.001, df = 1, *p* = 1.0, *n* = 166; Sex, LR *χ*^2^ = 0.079, df = 2, *p* = 0.96, *n* = 141). Also, the interaction between sex and wing length rank was not significant (GLM, LR *χ*^2^ = 0.85, df = 2, *p* = 0.65, *n* = 141). The body mass rank was not significantly related to nestling infection status (GLM, body mass rank, LR *χ*^2^ = 0.83, df = 1, *p* = 0.36, *n* = 141; sex, LR *χ*^2^ = 0.047, df = 2, *p* = 0.98, *n* = 141). Also here, the interaction between sex and body mass rank was not significant (GLM, LR *χ*^2^ = 0.33, df = 2, *p* = 0.85). The tarsus length rank showed a marginally significant interaction with sex (GLM, LR *χ*^2^ = 5.94, df = 2, *p* = 0.05, *n* = 141; Fig. 2, right panel).

### Comparison infection status of adults and their nestlings (Q9)

The 44 adults (12 males, 32 females) we screened during the 2014 breeding season belong to 32 broods with known parasite infection status (Table 1). All males and 23 females were infected with at least one parasite genus. Sixteen birds were infected with *Leucocytozoon*, three with *Plasmodium*, two with *Haemoproteus*, six with a mixed infection of *Leucocytozoon* and *Haemoproteus*, seven with *Leucocytozoon* and *Plasmodium,* and one with *Haemoproteus* and P*lasmodium*. Those 32 broods had 85 nestlings of which 35 were infected (33 with *Leucocytozoon*, 2 with *Haemoproteus)* and 50 were uninfected. There was no obvious pattern between infection status of the parents and their nestlings (Table 1). For nine broods where at least one parent and one nestling were infected, we also have information on the lineage. In six cases, nestling and adults both were infected with COCOR13. In the remaining three cases, lineages differed between adults and nestlings.

**Table 1:** Frequency table showing occurrence of different combinations of haemosporidian infection status of male and female jackdaw parents and their respective nestlings during the 2014 breeding season in a nest box colony in southern Sweden. Due to the unbalances/limited sample size, no statistical tests were applied

Infection status	All nestlings infected ●	Some nestlings infected ◖	All nestlings uninfected ○	(Total number of broods)
Both parents infected ●●	4	6	1	(11)
Male parent infected, ●○ female parent uninfected	0	0	1	(1)
Female parent infected ● (male parent unknown)	2	6	4	(12)
Female parent uninfected ○ (male parent unknown)	0	4	4	(8)

## Discussion

We found that jackdaw nestlings at the age of 29 days are commonly infected with blood parasites, with *Leucocytozoon* being the most prevalent genus detected. We additionally found bigger broods to have a higher likelihood of having at least one infected nestling. Among partially infected broods, nestling sex in combination with size rank within a brood were significantly related to nestling infection status. Between broods, however, we did not detect significant relationships between infection status and morphometric measurements, sex, or hatching date. There were also no obvious patterns relating infection rates of adults to their nestlings giving no evidence for transgenerational infections.

### Parasite prevalence, parasite genera, and lineage diversity

The differences in prevalence across the four study years may be caused by variations in ambient conditions. For instance, the month of May (i.e. the period when nestlings would have been bitten by vectors) was in 2015 about 0.5 °C colder than average (Sweden’s Meteorological and Hydrological Institute, SMHI 2024) and more windy than usual (AH, personal communication). The years 2014, 2018, and 2022, on the other hand, had warmer than average temperatures in May (SMHI 2024). Studies have found evidence for decreased dipteran abundance of vectors with higher wind speeds (Blackwell 1997; Kettle 1957; Martínez-de la Puente et al. 2009), and at lower temperatures (Martínez-de la Puente et al. 2009). An alternative explanation for the lower haemosporidian prevalence in 2015 could be selective mortality: If cold and windy conditions lead to reduced food availability for the birds (Starck and Ricklefs 1998), it is possible that nestlings with poor nutritional status and consequently poor immune function died before blood sampling (Christe et al. 1998), leading to a low apparent prevalence. And indeed, nestling survival in our jackdaw population was low in 2015 and we know from a detailed study done during the 2018 breeding season that birds which did not survive the nestling period had reduced immune function at day 12 compared to surviving birds (Aastrup et al. 2023).

The parasite genus *Leucocytozoon* constituted the majority of infections (95.2% of all infections, *n* = 119 out of 125). This may be related to genus-specific prepatent periods: According to Valkiūnas (2005), *Leucocytozoon* has a prepatent period of 4–15 days (*Plasmodium:* 2 days to 3 months; *Haemoproteus:* 14–38 days). It is possible that some *Plasmodium* and *Haemoproteus* infections were not yet detectable in the peripheral blood at the time of sampling as they were still in the prepatent stage, while Leucocytozoon infections acquired at the same age had already passed the prepatent period. Indeed, increases of prevalence with nestling age have already been found for all three haemosporidian genera (Townsend et al. 2018). Only studies targeting host species with even longer nestling periods could corroborate true prevalence differences. Additionally, it is worth noting that we only used molecular methods to detect infections and had no blood slides available for microscopic examination. Hence, we cannot rule out that some infections were missed.

In 2015, when overall haemosporidian prevalence was lowest, we found a similar number of different *Leucocytozoon* lineages. However, the dominant lineage COCOR13, made up a much lower proportion of infections than in other years, which could be caused by between year variations in vector composition driven by weather conditions (see above). It is worth noting that, even though more typically found in Paridae species, the lineage PARUS21, which we found in our jackdaws, has also been found in Accipitridae, Certhiidae, and Fringillidae in other studies (according to MalAvi database).

Overall, the fact that nestlings within a brood were infected by different lineages suggests they were likely bitten by different individual vectors. To identify potential vectors at the jackdaw study site, we carried out a pilot insect trapping in the nest boxes during the nestling rearing period of 2022 (see supplementary material for trapping method, design, and possible limitations). We found several biting midges of which one was engorged with a blood meal. All other insects on the traps are not known to transmit any haemosporidian parasites. In one nest box with a potential vector, brood infection status was unknown. In the other nest boxes with a potential vector, broods had *Leucocytozoon* infections. However, biting midges are known to transmit *Haemoproteus* rather than *Leucocytozoon* parasites, the vectors of which are suggested to be exophagous, i.e. feeding only outside of enclosed spaces (Votýpka et al. 2009). Yet, our results together with those by Chakarov et al. (2021) suggest these vectors regularly enter nest boxes, since we found a considerable prevalence of *Leucocytozoon* infections in the nestlings (32.8% of all nestlings).

### Hatching date and absolute body measurements

Haemosporidian prevalence was not related to hatching date in our study, which might be a consequence of the relatively synchronised breeding season of jackdaws. All nestlings reached the age of 29 days within a 14-day window (2nd to 16th June)—very short compared to a 72-day window in Common Buzzards and Sparrowhawks in the study by Svobodová et al. (2015). This suggests that such differences in prevalence caused by seasonality will only show in species with less synchronised breeding seasons, like raptors or non-colonial breeding passerines.

Neither body mass nor wing or tarsus length was correlated with infection status in our study at the population level. In line with the tasty chick hypothesis (Christe et al. 1998), we had expected higher prevalence in smaller nestlings because of their poorer immune function (Aastrup et al. 2023). Conversely, Carnid Flies (*Carnus haemapterus)* preferred the heaviest American Kestrel (*Falco sparverius*) nestlings up to 5 days of age (Dawson and Bortolotti 1997), in line with Valera et al. (2004), who have suggested that dipteran insects might prefer to bite larger nestlings, as they provide better resources*.* But compared to nest-dwelling ectoparasites, the vectors in our system need to detect active nest boxes to find and bite susceptible nestlings and it is unlikely for free-flying vectors to assess nestling size in a nest box system. Furthermore, the levels of vector attractants emitted by the nestlings might be more pooled within a cavity compared to open nest breeders and size differences between conspecific nestlings might be too small to have a significant impact (Gutiérrez-López et al. 2019).

### Parasite prevalence and body measurement ranks within brood

While we did not find infection to be related to biometric measurements across the study population, we found such evidence at the brood level. Specifically, among females, those with the shortest tarsi were significantly more likely to be infected than their female siblings with longer tarsi. This relationship at the brood level, while absent on the population level, supports the tasty chick hypothesis in a way that smaller individuals are at higher risk for infection. From a previous study in this jackdaw population, we know that male nestlings are larger than females and that smaller nestlings likely sit closer to the nest entrance (Aastrup and Hegemann 2021; Aastrup et al. 2023). Consequently, the smaller females sitting close to the nest box entrance could also have a higher risk of becoming infected even at the same age as male siblings as they may have a high encounter rate with vectors. An additional, but not mutually exclusive, hypothesis is that small (i.e. underdeveloped) nestlings may show less effective behavioural defences against dipterans and immune responses against infections (Garrido-Bautista et al 2022).

### Brood size and brood infection status: full, none, or partial

At the level of entire broods, presence of infection significantly increased with increasing clutch size. This is in line with a trend found in Sparrowhawk and Common Buzzard nestlings (Svobodová et al. 2015). A higher biomass of nestlings is expected to produce more of the attractants helping dipteran vectors to locate their hosts (Castaño-Vázquez et al. 2020). Likewise, larger broods of Great Tits (*Parus major*), Blue Tits (*Cyanistes caeruleus*), and Pied Flycatchers (*Ficedula hypoleuca*) attracted more black flies and biting midges (Martínez-de la Puente et al. 2009). Though, it is not clear if the effect we found is (i) truly a consequence of increased brood size or (ii) an artefact of larger broods having a higher chance of one or more nestlings becoming infected. For (i) also, the individual infection rate would increase along with increasing brood size, which was not the case in our study. Interestingly, Svobodová et al. (2015) found an opposite trend, with weakly but consistently decreasing prevalence along with increasing brood size, suggesting an encounter-dilution effect (Rätti et al. 2006).

A possible explanation for the proportion of partially infected broods in our study could be that vectors only collect one blood meal. If this would be true, multiple infected nestlings in a brood are a result of bites from several vectors, which is also supported by the fact that we found different haemosporidian lineages within broods. This contrasts with findings by Svobodová et al. (2015), where infection status and parasite genus were not independent for siblings within broods. However, we cannot rule out that there is a hidden lineage diversity in some hosts which was masked by bias in PCR and Sanger sequencing (see Pibaque et al. 2026).

### Growth rate

Infection status was statistically unrelated to growth rate in any of the three body size metrics. Even though we did not have the infection status at day 12 of age (but only at day 29 of age), the prepatent period of haemosporidians makes it likely that nestlings got infected at early age, likely around day 12. This justifies a comparison of growth rates, even though we could not distinguish between stages of infection which may mask differences and may become visible only at the highest levels of infection intensity (Rinaud et al. 2025). Our findings are in accordance with the other studies (Dunn et al. 2017; Hasselquist and Nilsson 2012; Rinaud et al. 2025) and suggest that the nestlings grow at the same rate whether they are infected with haemosporidians or not. As observed differences between infected and uninfected nestlings could either be a cause or an effect of infection, the observed relationship between tarsus length and infection in our female jackdaw nestlings might be a consequence of smaller female nestlings being more likely to get infected, rather than female nestlings growing slower as a result of being infected.

### Adult infection status

Similarities in infections between parents and their offspring could be expected if vectors bite parents and later their offspring. If this would happen, particularly the female parent and the offspring would have similar infections in our study as only the female parent incubates and broods the nestlings. However, when comparing infection status of adults and their nestlings, we detected no obvious pattern between infection status of the parents and their nestlings, which is in contrast to a study on Common Buzzards, where Chakarov et al. (2015) found evidence for vector-mediated parent-to-offspring transmission. One explanation could be the relatively close distance of our jackdaw broods to each other, compared to Buzzards, which makes it easy for non-specialised vectors such as blackflies to visit multiple broods (Chakarov et al. 2020). Even if the parasite lineage was the same for 66% of broods where at least one parent and one nestling were infected, those were all infections with COCOR13, the most prevalent lineage in every study year. Hence, the same lineage for parents and offspring can just as well be a result of bites by two different vector individuals carrying this very abundant parasite lineage.

## Conclusion and outlook

We found some patterns of blood parasitism in jackdaw nestlings, that hint at complex relationships between host, vector, and parasite. In particular, brood infection status increased with brood size, while individual infection did not, which is consistent with combinatorial probability and/or greater attractants in larger broods. We also found some support for the tasty chick hypothesis in haemosporidian infections, while it was originally developed for ectoparasites. Furthermore, a high frequency of partially infected broods as well as the occurrence of more than one lineage within broods suggests multiple vectors biting, but further studies are required. Finally, the pronounced year-to-year variation suggests that the vector community may be under strong environmental influence. Linking environmental data to infection status was beyond the scope of this study and hence requires further testing. Taken together, the patterns of prevalence for haemosporidians are always the result of vector choices and the interactions between the parasites and host characteristics. With our study, we have taken the first steps towards a more comprehensive understanding of the infections in nestlings, and we hope that our study will inspire more research on the complex host–vector–parasite relationships of haemosporidian infections in nestling birds.

## Supplementary Information

Below is the link to the electronic supplementary material.

Supplementary file1 (DOCX 3297 KB)
