# Supplementary material for: Within- and between-brood variations in haemosporidian infections of nestlings reveal complex host–vector–parasite relationships
Source: J Ornithol. 2026 Jun 3;167(3):803–16. doi: 10.1007/s10336-026-02417-1 (PMC13342419; doi:10.1007/s10336-026-02417-1)
Supplement: Supplementary file 1 — Supplementary file1 (DOCX 3297 KB) [file 10336_2026_2417_MOESM1_ESM.docx]

**Electronic Supplementary Material**

**Within- and between-brood variations in haemosporidian infections of nestlings** **reveal complex host-vector-parasite relationships**

Anna George^1^, Christian Aastrup^1^, Tamara Emmenegger^1,2^, Arne Hegemann^1,3*^

^1^ Department of Biology, Lund University, Ecology Building (Sölvegatan 37), 223 62, Lund, Sweden

^2^ Museum Lucerne, Department of Zoology, Kasernenplatz 6, 6003 Lucerne, Switzerland

^3^ Institute of International Animal Health / One Health, Friedrich-Loeffler-Institute, Federal Research Institute for Animal Health, Südufer 10, 17493 Greifswald - Insel Riems, Germany

*Corresponding author: [arne.hegemann@fli.de](mailto:arne.hegemann@fli.de)

**Method for vector trapping**

To identify haemosporidian vectors at the study site, we carried out vector trapping in the nestboxes during nestling rearing in 2022, using a method adapted from Tomás et al. (2008) and Castaño-Vázquez et al. (2022). Sticky cards were placed on 31st May 2022 in 23 nest boxes in which nestlings were between 12-22 days old and collected 4 days later. Based on a pilot study we chose to use unscented white petroleum jelly (a.k.a. Vaseline) as this had the best lasting stickiness and was neutral to vectors (i.e. not attracting or repelling). Sticky cards consisted of a rectangle of water-resistant card measuring 12 x 25 cm, with 5ml of petroleum jelly spread evenly over the surface and pinned with four thumbtacks to the inner roof of the nest box. Given that we placed our sticky cards relatively late during the nestling period, leaving only a short period between vector catching and blood sampling to screen for haemosporidian infections, we may have missed direct links due to the prepatent period.

**Table S1** Frequency table showing numbers of jackdaw nest boxes in which potential vectors for haemosporidian infections were found, together with numbers of nest boxes in which at least one nestling was infected with a haemosporidian parasite.

|  | Infection yes ● | Infection no ○ | (Total) |
| --- | --- | --- | --- |
| Vectors yes ● | 5 | 0 | (5) |
| Vectors no ○ | 8 | 7 | (15) |
| (Total) | (13) | (7) | (20) |

**Figure S1**: Map of the Jackdaw study colony at Revinge, southern Sweden. Nestbox positions as shown on the map are taken with GPS and depending on GPS accuracy might deviate by a few meters from real positions.


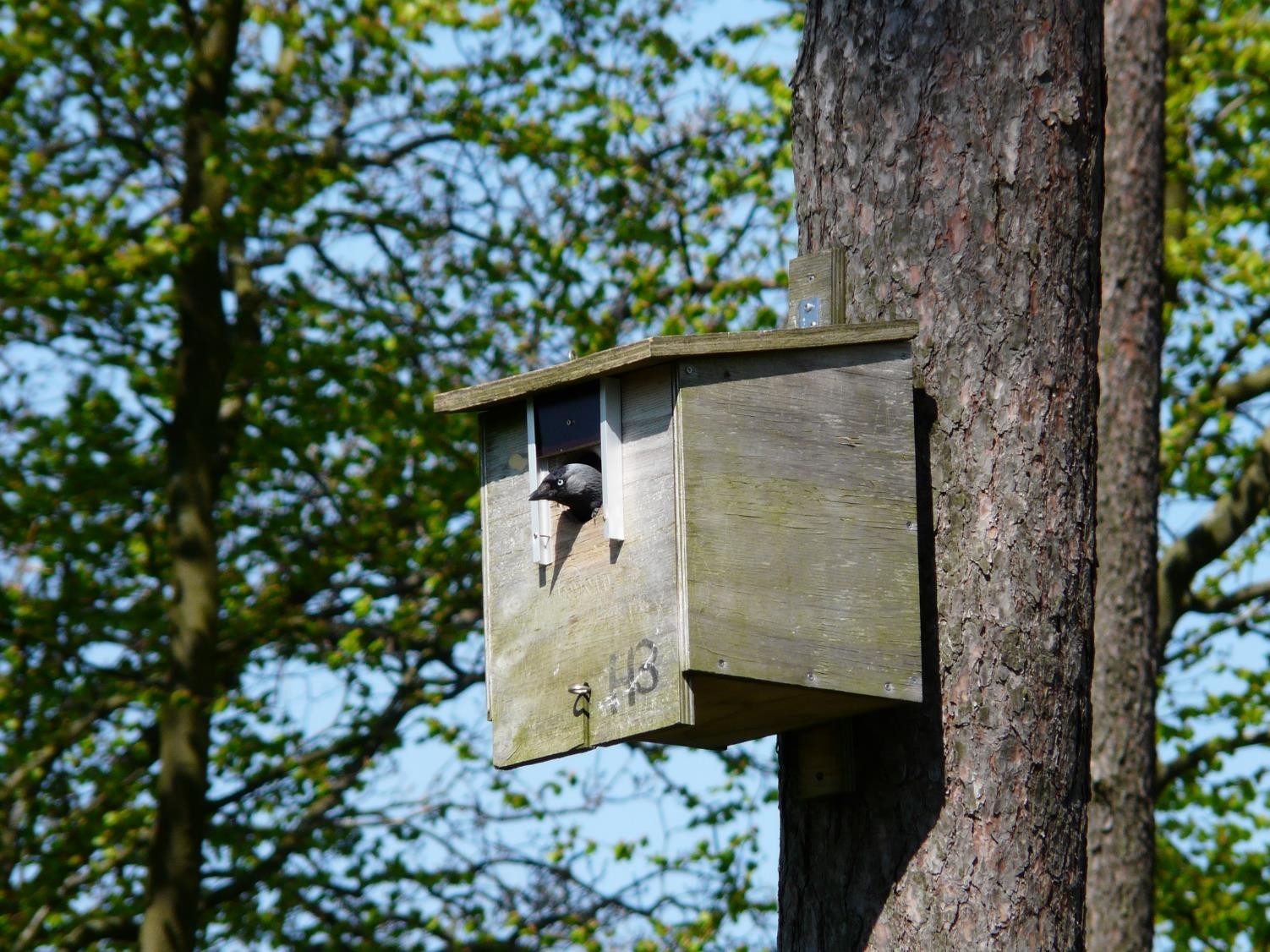


**Figure S2**: Example of a Jackdaw nestbox in the study colony at Revinge, southern Sweden.
